# Revisiting the Dissolution of Cellulose in NaOH as “Seen” by X-rays

**DOI:** 10.3390/polym12020342

**Published:** 2020-02-05

**Authors:** Birte Martin-Bertelsen, Erika Andersson, Tobias Köhnke, Artur Hedlund, Lars Stigsson, Ulf Olsson

**Affiliations:** 1 Division of Physical Chemistry, Lund University, 221 00 Lund, Sweden; 2 Division Materials and Production, RISE Research Institutes of Sweden, 431 53 Mölndal, Sweden; 3 KIRAM AB, Norra Villavägen 17, 237 34 Bjärred, Sweden

**Keywords:** cellulose dissolution, microcrystalline cellulose (MCC), cold alkali (NaOH), ZnO, poly(ethylene glycole) (PEG), small-angle X-ray-scattering (SAXS), static light scattering (SLS)

## Abstract

Cotton production is reaching a global limit, leading to a growing demand for bio-based textile fibers produced by other means. Textile fibers based on regenerated cellulose from wood holds great potential, but in order to produce fibers, the components need to be dissolved in suitable solvents. Furthermore, the dissolution process of cellulose is not yet fully understood. In this study, we investigated the dissolution state of microcrystalline cellulose in aqueous NaOH by using primarily scattering methods. Contrary to previous findings, this study indicated that cellulose concentrations of up to 2 wt % are completely molecularly dissolved in 8 wt % NaOH. Scattering data furthermore revealed the presence of semi-flexible cylinders with stiff segments. In order to improve the dissolution capability of NaOH, the effects of different additives have been of interest. In this study, scattering data indicated that the addition of ZnO decreased the formation of aggregates, while the addition of PEG did not improve the dissolution properties significantly, although preliminary NMR data did suggest a weak attraction between PEG and cellulose. Overall, this study sheds further light on the dissolution of cellulose in NaOH and highlights the use of scattering methods to assess solvent quality.

## 1. Introduction

**Figure 1:**
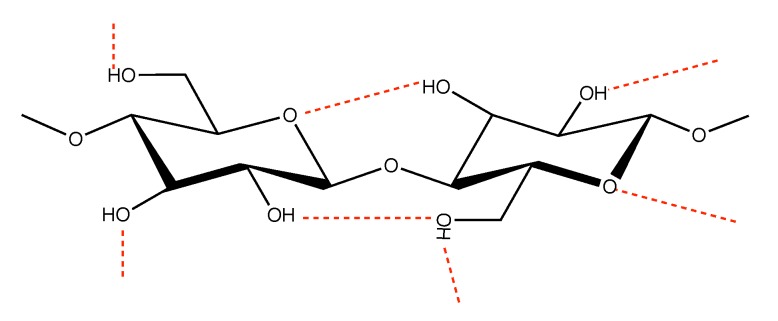
Molecular structure of cellulose.

Cotton fibers are eminent as textile fibers, but cotton production is reaching a global limit, mainly due to environmental considerations [1]. Since the main component of cotton is cellulose (90%) [2], there has been a strong focus on the prospect of utilizing regenerated cellulose from other sources, such as wood, to produce textile fibers. Examples of textile fibers based on regenerated cellulose are viscose and lyocell [3]. In order to produce textile fibers from wood-based sources, the components need to be dissolved in a suitable solvent. Cellulose (Figure 1) is a semi-crystalline polymer [4] known to be insolvable in most common polar solvents [5]. The underlying mechanisms of cellulose dissolution have not yet been fully understood, but the involvement of both hydrogen bonding and hydrophobic interactions have been discussed in literature [5,6,7,8]. In this context it has also been noted that the overall kinetics of dissolution are governed by the balance of the two inter-related phenomena of decrystallization and chain disentanglement [9].

Substantial effort has been put toward identifying suitable solvents. One of the most well-studied of these is NaOH, which from an industrial perspective is both cheap and environmentally friendly. One of the challenges of using aqueous NaOH as a solvent for cellulose is that cellulose is only dissolved within a narrow range of NaOH concentrations (1.5–2.5 M NaOH) and at temperatures below 0 ℃ [5,10]. Furthermore, the amount of cellulose that can be dissolved in neat NaOH is low, and previous studies differ on the reported maximum concentration of cellulose that can be dissolved in neat NaOH. For microcrystalline cellulose (MCC), the reported values for complete dissolution range from 1 wt % up to 4–6 wt % [11,12]. By using shear rheology it has also been found that MCC in 9 wt % NaOH is characterized as a suspension with aggregate-based gelation occurring at MCC concentrations above 1.5 wt % [13].

In order to improve the dissolution capability of NaOH, the effects of adding different additives to aqueous NaOH have been of interest. Amongst others, ZnO, urea and PEG have in the literature been reported to positively affect the dissolution properties of NaOH [5]. In this study the goal was to investigate cellulose in aqueous NaOH with and without additives in order to investigate the dissolution state; i.e., whether the cellulose is completely dissolved at a molecular level or aggregates are still present. Small-angle X-ray scattering (SAXS) and static light scattering (SLS) were employed in order to investigate the samples at a molecular level, and to compare the levels of aggregation present. Furthermore, a section is included in this paper that explores the guiding principles for the dissolution of cellulose in NaOH in order to highlight relevant considerations, which may be of use in order to improve cellulose processing. In our group at Lund University, we have previously employed scattering methods to study the dissolution properties of cellulose in aqueous NaOH [11], and in aqueous tetrabutylammonium hydroxide (TBAH) [14,15,16]. In the present study the main purpose was to utilize scattering techniques in order to qualify and compare the dissolution properties of different additives with respect to neat NaOH. Thus, this study also demonstrates the usefulness of scattering techniques in the process of assessing different solvent systems.

## 2. Materials and Methods

### 2.1. Materials

Microcrystalline cellulose (MCC) was obtained from Sigma-Aldrich, Merck KGaA, Darmstadt, Germany (Avicel PH-101, average particles size of 50 μm). Sodium hydroxide, 97% pure, as anhydrous pellets, was purchased from Merck KGaA, Darmstadt, Germany. The water used was purified in-house using a MILLIPORE Milli-Q Gradient A 10 (Millipore, Molsheim, France). Poly(ethylene glycol) (PEG) with an average molecular weight of 200 g/mol was obtained from Sigma-Aldrich, Merck KGaA, Darmstadt, Germany. Zinc oxide, 99.99% pure, was obtained from Alfa Aesar/ThermoFisher, Kendel, Germany. Sodium deuteroxide 40 wt % in D_2_O, 99.5% D and D_2_O was obtained from Sigma-Aldrich, Merck KGaA, Darmstadt, Germany.

### 2.2. Sample Preparation

MCC was dissolved in 8 wt % (2 M) aqueous NaOH by using a freeze-thaw method: MCC was added to NaOH(aq) at 0 ℃ while stirring vigorously for 15–20 min (samples kept in an ice bath). The samples were subsequently left in the freezer at –18 ℃ overnight and next day thawed in an ice bath while being stirred for approximately 20 min. If applicable, additives were added to NaOH(aq) before the addition of MCC. Since the solubility of ZnO is strongly dependent on pH, ZnO was solubilized at higher NaOH concentrations (18–20 wt %), and afterwards the solutions were diluted to obtain 8 wt % NaOH. NMR samples were prepared similarly, but by using NaOD and D_2_O.

### 2.3. Static Light Scattering (SLS)

Static light scattering experiments were performed using a 3D LS Spectrometer (LS-Instruments AG, Fribourg, Switzerland) equipped with a laser producing a photon beam with a wavelength of 660 nm. The data were obtained over an angular range of 60–140° with a step size of 1°. Exposure time was 10 × 30 s per step. The temperature was kept at 25 ℃, controlled by a circulating water bath (Julabo, Seelbach, Germany). The scattering intensity of the neat 8 wt % NaOH solution was subtracted as background, and the scattering data were converted to absolute scale, i.e., the excess Rayleigh ratio ΔR(q), according to [17]:

(1) $$\Delta R\left(q\right)=\frac{\Delta I(q)}{I_{\mathrm{tol}}\left(q\right)}{\left(\frac{n}{n_{\mathrm{tol}}}\right)}^{2}R_{\mathrm{tol}},$$

where $I_{\mathrm{tol}}\left(q\right)$, $n_{\mathrm{tol}}$ and $R_{\mathrm{tol}}$ correspond to the scattered intensity, refractive index and Rayleigh ratio of the reference toluene. The scattering vector *q* is defined by q=4πnsinθ/λ, depending on the refractive index *n*, the wavelength λ and the scattering angle given by 2θ. The mean intensity and corresponding standard deviations (SDs) were calculated from the 10 measurements at each step.

### 2.4. Refractometry

Refractive indices were determined by using an Abbe 60 Refractometer (Bellingham and Stanley Ltd., Kent, UK). The temperature was kept at 25 ℃, controlled by a circulating water bath (Julabo, Seelbach, Germany). The refractive indices for different concentrations of MCC were determined at three different wavelengths; 435.8, 546.1 and 579.0 nm. The refractive indices at 660 nm were obtained through Cauchy’s empirical equation:

(2) $$n\left(\lambda \right)=A+\frac{B}{\lambda^{2}},$$

where λ denotes the wavelength and *A* and *B* are sample-specific coefficients determined by fitting the equation to the measured refractive indices.

### 2.5. Small-Angle X-ray Scattering (SAXS)

Small-angle X-ray scattering measurements were performed using the laboratory-based SAXSLab Ganesha 300XL instrument (SAXSLAB ApS (Xenocs), Skovlunde, Denmark) equipped with an X-ray source producing a photon beam with a wavelength of 1.54 Å. The scattering patterns were recorded with a two-dimensional (2D) 300 K Pilatus pixel area detector (Dectris, Baden, Switzerland). The samples were measured at a temperature of 25 ℃, which was controlled by an external recirculating water bath (Julabo, Seelbach, Germany). The 2D scattering data did not show any angular dependency, and were thus azimuthally averaged; normalized by the incident radiation intensity, the sample exposure time and transmission; and corrected for background and detector inhomogeneities. The radially averaged intensity *I* is given as a function of the scattering vector q=4πsinθ/λ, where λ is the wavelength and 2θ is the scattering angle. The samples were measured at three settings covering a *q*-range from approximately 0.004 to 3 1/Å. Data were brought to absolute scale using the SAXSGui software provided by the instrument. In order to assess the scattering intensity of MCC, $I_{\mathrm{MCC}}$, the scattering intensity of the solvent, I(s+c), and the empty capillary, I(c), were subtracted from the experimentally determined scattering intensity, I(MCC+s+c), using the volume fraction, $\phi_{\mathrm{MCC}}$, of MCC:

(3) $$I_{\mathrm{MCC}}=I\left(\mathrm{MCC}+s+c\right)-\left(1-\phi_{\mathrm{MCC}}\right)I\left(s+c\right)-\phi_{\mathrm{MCC}}I\left(c\right).$$

The respective volume fractions were calculated from the weight fractions by using the density of MCC, $\rho_{\mathrm{MCC}}=1.5$ g/cm${}^{3}$, and the respective densities of NaOH and the additives studied. Data analysis and simulations of theoretical model fits were performed using MATLAB (The MathWorks, Inc., MA, USA) and SasView (www.sasview.org [18]).

### 2.6. NMR Diffusion

NMR diffusion experiments were performed on a Bruker Avance DMX200 spectrometer (Bruker, Billerica, MA, USA) operating at 200.13 MHz on the ${}^{1}$H nuclei, equipped with a commercial diffusion probe (DIF-25 5 mm) and a maximum gradient strength *G* of 960 *G* 1/cm. Measurements were done at 25 ℃ using dry air as the temperature control gas. Pulsed gradient stimulated echo with a field gradient pulse of δ=2 ms, a separation of Δ=140 ms and a varying gradient strength of G=5.8–25 *G* 1/cm was used to measure water diffusion in samples of 2M NaOD and 0.1 or 1 wt % PEG-200 at different concentrations of MCC. The diffusion of PEG in the 0.1 wt % PEG samples was obtained in the same way, but with a varying gradient strength of G=12.5–50 *G* 1/cm. Non-stimulated pulsed spin echo was employed for the 1 wt % PEG samples in order to detect the sharp PEG peak and not the overlapping broad peak from cellulose (otherwise with the same parameters). The diffusion coefficients *D* were calculated using the software Topspin 4.0.6 by fitting an exponentially decaying function to the measured echo amplitude *I*

(4) $$I=I_{0}\;e^{-bD},$$

were $I_{0}$ is the amplitude at G=0 and

(5) $$b={\left(\gamma \;G\;\delta \right)}^{2}\left(\Delta -\delta /3\right),$$

were γ is the magnetogyric ratio [19,20].

## 3. Results and Discussion

### 3.1. Revisiting Cellulose in 2 M NaOH(aq) at 25 ℃

**Figure 2:**
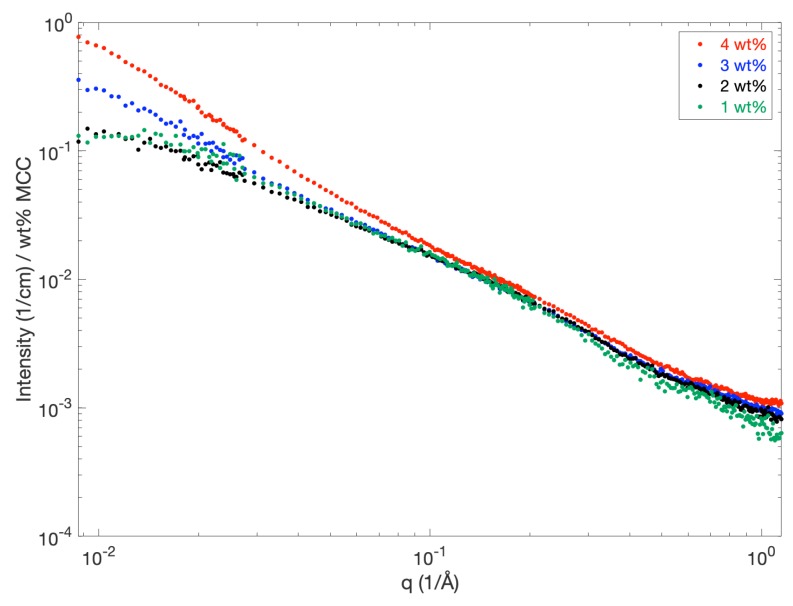
SAXS curves of microcrystalline cellulose (MCC) dissolved in 8 wt % (2 M) NaOH for MCC concentrations of 1, 2, 3 and 4 wt % MCC. The scattered intensity was scaled with the respective MCC weight fractions to facilitate comparisons of the different scattering curves.

Different concentrations of MCC dissolved in NaOH were studied by using SAXS. A comparison of the SAXS curves for MCC concentrations of 1, 2, 3 and 4 wt % is shown in Figure 2. Note that the scattered intensity has been scaled by the MCC concentration in order to facilitate comparisons between the scattering curves. At high *q*, the scattering intensity is similar for all four MCC concentrations. At low *q*, the scattering intensity of 1 and 2 wt % MCC seems to level off at approximately $1.5\times 10^{-1}$, while an upturn in intensity is observed for MCC concentrations of 3 and 4 wt %. Since the samples containing 3 and 4 wt % gelled much faster compared to the 1 and 2 wt % ones, the increased scattering intensity at low *q* can be ascribed to the presence of aggregates/branched cylinders in the solution.

The scattering patterns obtained for 1 and 2 wt % MCC are similar in the whole *q*-range, which indicates that cellulose is completely dissolved at these concentrations. This is in contrast to previous observations reported by Hagman et al. [11], where the scattering pattern of a solution containing 1 wt % MCC revealed an approximately 10-fold higher scattering intensity at low *q*. Slight changes in the sample preparation protocol (i.e., longer freezing time or longer stirring time in ice bath) are most likely the cause of the observed difference in dissolution and highlight the sensitivity of the cellulose-NaOH-system with respect to these parameters.

As already discussed by Behrens et al. [14], for MCC dissolved in TBAH, a possible explanation for the increase in scattering intensity for MCC concentrations above 2 wt % can be found by considering the solubility of cellulose I versus cellulose II. Cellulose II corresponds to the non-native, regenerated form of cellulose with anti-parallel chain ordering. Cellulose II is more stable and has a lower chemical potential compared to cellulose I, and thus the solubility of cellulose II is lower compared to cellulose I. Thus, at MCC concentrations above 2 wt %, the solution may consist of dissolved cellulose I and a supersaturation with respect to cellulose II, which will precipitate and give rise to an increase in the scattering intensity. At MCC concentrations below 2 wt %, most likely both cellulose I and II can be dissolved, but after prolonged storage at room temperature, these solutions are also known to form gels [21].

**Figure 3:**
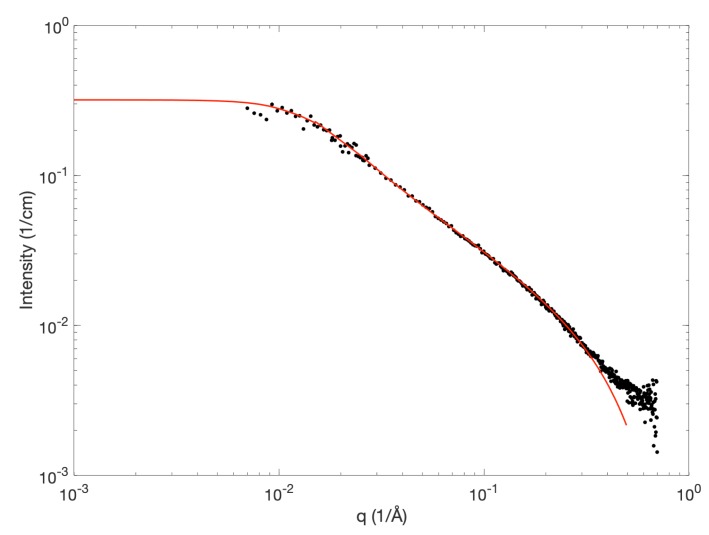
Experimental data for 2 wt % MCC in 8 wt % NaOH (black dots) together with the theoretical scattering profile of semi-flexible cylinders (red curve). The model parameters used for the semi-flexible cylinder scattering profile were a radius of 4.1 Å, a persistence length of 100 Å and a total contour length of 600 Å.

To further investigate the state of dissolution at low wt % MCC, we focused on the X-ray scattering pattern for 2 wt % MCC in 8 wt % NaOH (Figure 3). The slope for *q*-values in the range of approximately $10^{-2}<q<10^{-1}$ 1/Å was determined to be 1/q, which indicates the presence of rod-like scattering objects. The scattered intensity as a function of the scattering vector *q* can be expressed as

(6) $$I\left(q\right)=KcM_{w}P\left(q\right)S\left(q\right),$$

where *K* is the optical constant depending on the type of scattering technique, *c* is the concentration of the solute, $M_{w}$ is the weight-averaged molecular weight, P(q) is the average form factor of the polydisperse cellulose distribution and S(q) is the effective structure factor.

Due to the slope of 1/q, the theoretical form factor scattering profile of semi-flexible cylinders with excluded volume effect was plotted against the experimental data (red curve, Figure 3). The model describes the scattering of semi-flexible cylinders with a radius *r* and a total contour length *L*, consisting of stiff segments with lengths given by a persistence length $l_{p}$[18]. The persistence length is related to the Kuhn length $l_{k}$ by $l_{k}=2\;\cdot \;l_{p}$. In order to include the excluded volume effects due to the different chains, a structure factor described by the random phase approximation (rpa) was included in the model

(7) $$S\left(q\right)=\frac{1}{1+\nu P(q)},$$

where the interaction parameter ν is given by ν=1/S(0)−1.

The parameters applied in the displayed model where: ν=1, c=0.02 g/cm${}^{3}$ and *K* given by

(8) $$K_{\mathrm{SAXS}}=\frac{\Delta \varrho^{2}}{\rho^{2}N_{A}},$$

where $N_{A}$ denotes the Avogadro constant, the mass density ρ of cellulose is ρ=1.5 g/cm${}^{3}$ and the scattering length difference Δϱ of cellulose in NaOH is calculated to be $\Delta \varrho =3.4\times 10^{10}$ cm${}^{-2}$. Furthermore, the $M_{w}$ used in the model simulations was calculated from an average DP value for Avicel PH101 estimated to be 218.2±6.9[22], which corresponds to an approximate average $M_{w}≃35.3$ kg/mol, assuming 162 g per anhydrous glucose unit.

The model fit depicted in Figure 3 corresponds to the scattering from cylinders with a radius of 4.1 Å, a contour length of 600 Å and a persistence length of 100 Å, indicating fibers with rather large, stiff segments. Previously, the persistence lengths of cellulose in various solvents have been experimentally determined to be in the range of 110–160 Å depending on the solvent system [23]. Specifically, for cellulose in 8 wt % NaOH, as determined by SLS, a persistence length of 110 Å has been reported [23,24], which is in good agreement with the persistence length of approximately 100 Å determined in the current study. Furthermore, a molecular dynamics simulation on cellulose in an aqueous solution, including solvent effects, revealed an upper limit for the persistence length of 145 ± 10 Å in aqueous solution [23]. A persistence length of 100 Å implies that the stiff segments 184 consist of approximately 19 anhydroglucose units (AGUs) based on the effective length of an AGU in solution determined to be 5.15 Å [25]. Thus, the chains must be sterically hindered, which could be due to solvent interactions or folded chain conformations. In the study by Kroon-Batenburg et al. [23] it is illustrated by the use of simulations that a $l_{p}$ for cellulose in the range of 90–125 Å indicates that up to 3% of folded conformations may occur. The determined cylinder radius seems to be in good agreement with the reported size of the glucose ring, which was calculated to be 8.6/8.4 Å for the long versus short axis of the molecule in the least hindered conformation [26].

As discussed above, the scattering intensity at low *q* is significantly lower than the scattering intensity observed by Hagman et al. [11], where the applied model was expanded with the theoretical scattering of cellulose “clusters” in order to capture the experimentally determined data. This could point towards the presence of aggregates in the previous study, which were not observed in this study. As mentioned above, this is most likely due to slight changes in the preparation protocol. Interestingly, in the study by Hagman et al. [11] the average apparent hydrodynamic radius, as determined by using pulsed gradient NMR on the self-diffusion of cellulose, was reported to be 33.6 nm, which was in disagreement with the scattering data [11], but seems to fit better with the observations reported in this study. Furthermore, the level of absolute scattered intensity at low *q* for 2 wt % MCC in NaOH is quite comparable to the reported scattered intensity at low *q* for 2 wt % MCC dissolved in TBAH, which was found to be close to a θ-solvent for cellulose (cf. Figure 3 in Behrens et al. [14]).

At *q*-values above $4\times 10^{-1}$, the model form factor of a semi-flexible cylinder starts to deviate from the experimental data. This might be explained by solvent-cellulose interactions that are not captured by the applied simple model. In order to completely capture this feature, it might not be sufficient to simply subtract the background solvent scattering as done in this study.

#### Static Light Scattering

**Figure 4:**
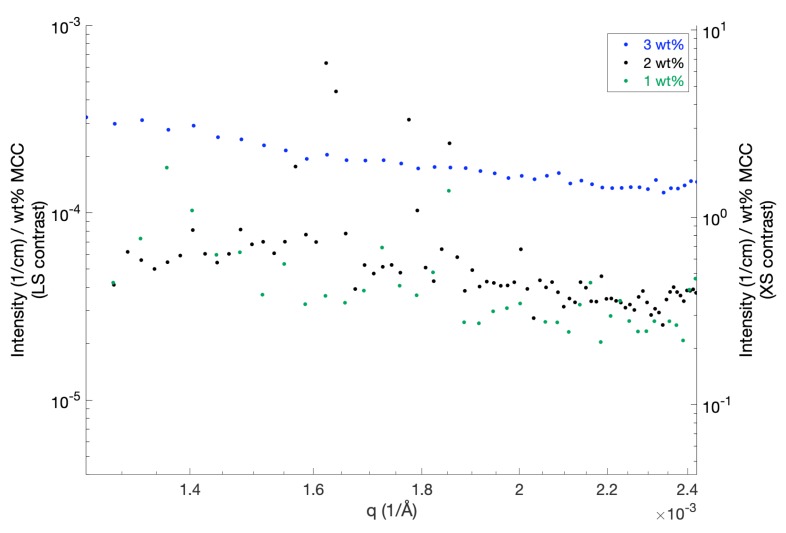
Static light scattering data for MCC dissolved in 8 wt % NaOH for MCC concentrations of 1, 2 and 3 wt %. The scattered intensity is displayed as original light scattering contrast (y-axis, left-hand side) versus scaled to SAXS contrast (y-axis, right-hand side). The intensity was, furthermore, scaled with the respective MCC weight fractions to facilitate comparisons of the different scattering curves.

To investigate further the scattering intensity at low *q*, a static light scattering study was performed on different concentrations of MCC in 8 wt % NaOH (Figure 4). The scattering intensities have been scaled with the respective MCC weight fractions in order to facilitate comparisons. In order to scale with the concentration, interactions between the particles have to be negligible, and this seems to be the case for the 1 and 2 wt % solutions. The scattered intensity in the whole *q*-range is similar for the solutions containing 1 and 2 wt % (directly proportional to the concentration), which is in good agreement with the SAXS observations, indicating that MCC is completely dissolved at these concentrations. At MCC concentrations above 2 wt %, the increased scattering intensity observed by using SLS (Figure 4) supports the formation of aggregates, as observed in the SAXS study.

In order to compare the scattering profiles with the scattering profiles obtained by using SAXS, the SLS data have been converted to SAXS contrast (scattering due to variations in electron density) by scaling with the optical constants for SAXS and SLS, $K_{\mathrm{SAXS}}/K_{\mathrm{SLS}}$ (Figure 4, y-axis right-hand side). $K_{\mathrm{SAXS}}$ is given by Equation (8). The optical constant for SLS is given by

(9) $$K_{\mathrm{SLS}}=\frac{4n_{0}^{2}\pi^{2}}{N_{A}\lambda^{4}}{\left(\frac{dn}{dc}\right)}^{2},$$

where $n_{0}$ is the solvent refractive index, λ denotes the photon wavelength and dn/dc corresponds to the refractive index increment. We determined the refractive index of the solvent and the refractive index increment by measuring the refractive indexes of different concentrations MCC in 8 wt % NaOH, yielding $n_{0}=1.3518$ (at λ=660 nm) and dn/dc≃0.113 cm${}^{3}$/g, respectively. The SLS scattering pattern revealed an increased scattering intensity compared to the SAXS scattering intensity at low *q*. This might indicate that a small fraction of large particles in the μm-range is present in the samples, which cannot be detected by SAXS. As mentioned by Burchard in Glasser et al. [8], the presence of a certain (small) amount of aggregates, which remain stable in dispersion, is intriguing. Filtering the samples may remove some of the larger aggregates, but even though sample filtration was employed in the study of Behrens et al. [14], the level of absolute intensity obtained in the present study for 1 and 2 wt % (MCC completely dissolved) is comparable to the reported absolute intensity obtained for 1 wt % MCC in TBAH (MCC completely dissolved). The detected sudden intensity fluctuations may indicate the presence of dust particles, and problems with these intensity spikes have been described before for filtrated solutions of dissolved MCC in TBAH [14].

A quick estimation of the observed effective $R_{g}$ can be determined by using the Guinier approximation $R\left(q\right)=R\left(0\right)\exp\left(-1/3\;R_{g}^{2}q^{2}\right)$. This gives the estimate $R_{g}\approx 58$ nm, which as expected, is significantly larger compared to the $R_{g}$ of approximately 11.3 nm calculated from the total contour length, *L*, and the persistence length, $l_{p}$, determined in the SAXS analysis by using the relation [23]

(10) $$\langle R_{g}^{2}\rangle =\frac{1}{3}Ll_{p}-l_{p}^{2}+2\frac{l_{p}^{3}}{L}-2\frac{l_{p}^{4}}{L^{2}}\left(1-\exp\left(-L/l_{p}\right)\right).$$

Similarly, an estimate of the observed weight-averaged molecular weight $M_{w}$ was determined from the Guinier approximation by using $R\left(0\right)=K_{\mathrm{SLS}}cM_{w}$ (assuming negligible interactions), which corresponds to a $M_{w}\approx 95$ kg/mol. This is also significantly larger than expected ($M_{w}\approx 35.3$ kg/mol; cf. Section 3.1), but a similarly large value ($M_{w}\approx 93$ kg/mol) has been determined previously for dissolved MCC in TBAH [14].

### 3.2. The Effects of Additives on the Dissolution Properties of Cellulose in NaOH

Several studies on additives, which can improve the dissolution properties of MCC in NaOH, have been reported in the literature [5]. ZnO has been identified as a promising candidate [27], but PEG has also been mentioned in that context [28] and holds great potential, since it is well-studied, cheap and harmless. The focus in the current study was to study and compare the effects of both ZnO and PEG by using SAXS to assess the dissolution quality. Since this study on MCC in NaOH demonstrated that it was possible to fully dissolve up to 2 wt % MCC (cf. Section 3.1), a SAXS study was performed on 4 wt % MCC in 8 wt % NaOH with and without the selected additives. A SAXS study on representative concentrations of PEG and ZnO added to 2 wt % MCC in 8 wt % NaOH did reveal similar scattering patterns at low *q*, which supports the finding that MCC is fully dissolved at this concentration (SI, Figure S1).

#### 3.2.1. ZnO

**Figure 5:**
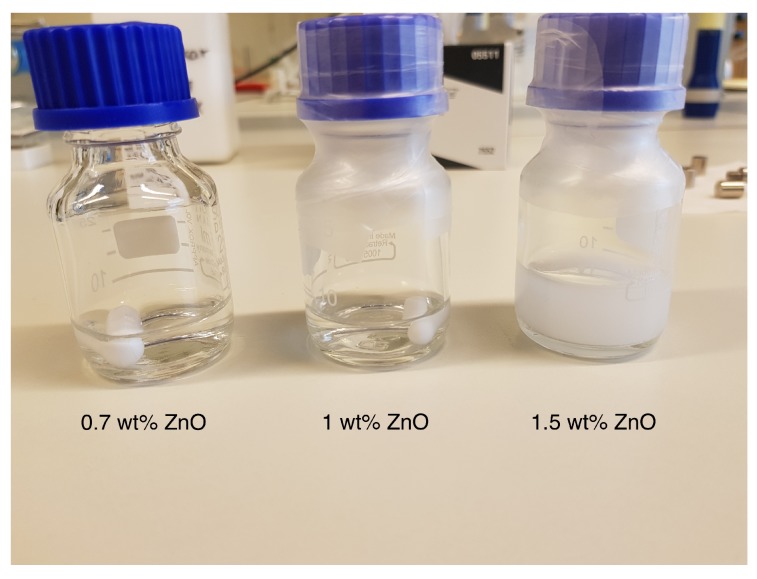
Different concentrations of ZnO in 8 wt % NaOH.

The solubility of ZnO is strongly dependent on the pH of the solution and has to be solubilized at a higher NaOH concentration and subsequently diluted to 8 wt %. Sedimentation of ZnO in 8 wt % NaOH has been previously observed when the concentration of ZnO is above 0.8 wt % [27]. In the current study, a ZnO concentration of 1.5 wt % resulted in a milky solution with particle sedimentation (Figure 5).

**Figure 6:**
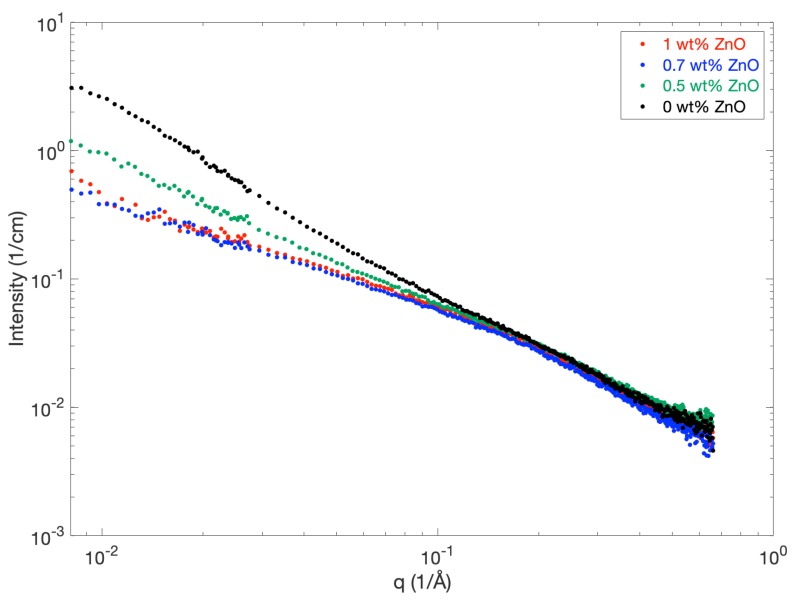
SAXS patterns for 4 wt % MCC dissolved in 8 wt % NaOH with/without a varying content of ZnO.

The dissolution properties of MCC in NaOH have been reported to improve when adding 0.5–2 wt % ZnO [5]. In order to study the effect of the ZnO concentration on the particle formation and the level of aggregation, SAXS measurements were performed on different samples containing 4 wt % MCC in 8 wt % NaOH and a varying ZnO content (Figure 6). The scattering experiments revealed a reduced scattering intensity at low *q* for the ZnO samples (red, blue and green curves) compared to MCC in NaOH without ZnO (black curve). This indicates that the addition of ZnO reduces the formation of aggregates, which fits well with previously reported observations that the addition of ZnO improves the dissolution by acting as a stabilizer against gelation [27,29]. The scattering data furthermore revealed that ZnO concentrations of 0.7 and 1 wt % seemed to have a similar effect on the level of aggregation (red and blue curves, Figure 6), whereas the addition of 0.5 wt % did decrease the level of aggregation to a lesser extent (green curve). Based on the level of aggregation, the current study, thus, indicates that an optimal ZnO concentration for 4 wt % MCC in 8 wt % NaOH is less than or equal to 0.7 wt % ZnO, but above 0.5 wt %.

If scaled with the respective MCC wt %, the absolute level of intensity at low *q* for the 1 and 0.7 wt % ZnO samples is similar to the absolute intensity obtained for completely dissolved MCC in neat NaOH (2 wt % MCC, Figure 2), although the curvature is slightly different. This could imply a change in fiber properties such as length and stiffness, but that was not further pursued in this study.

#### 3.2.2. PEG

**Figure 7:**
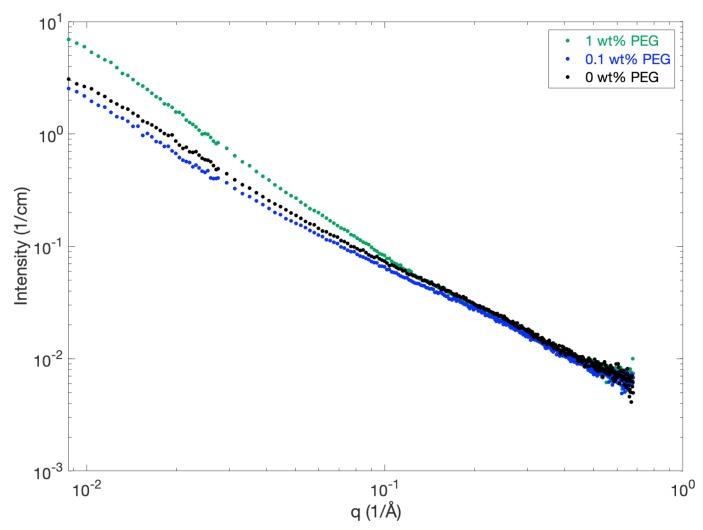
SAXS patterns for 4 wt % MCC dissolved in 8 wt % NaOH with/without a varying content of PEG with an average molecular weight of 200 g/mol.

The addition of PEG with an average molecular weight of 2000 g/mol has been reported to improve the dissolution properties of MCC in NaOH [28]. In the current study, PEG with a lower average molecular weight of 200 g/mol was selected due to problems with the solubility of PEG-2000 in NaOH (phase-separation occurred). The scattering curves for 4 wt % MCC in 8 wt % NaOH and different concentrations of PEG-200 are displayed in Figure 7. The scattering intensity at low *q* is slightly reduced for samples containing 0.1 wt % PEG (blue curve) compared to samples containing no additives (black curve), which suggests a slight decrease in the formation of aggregates. At a higher PEG content, the scattering intensity at low *q* is increased as compared to 4 wt % MCC in neat NaOH, indicating that the formation of aggregates is increased. Thus, only a low content of PEG-200 seems to slightly improve the dissolution properties of MCC in NaOH. However, the observed difference is so small that it is questionable whether this decrease in aggregation results in any noticeable difference of the properties on the macro-scale.

**Figure 8:**
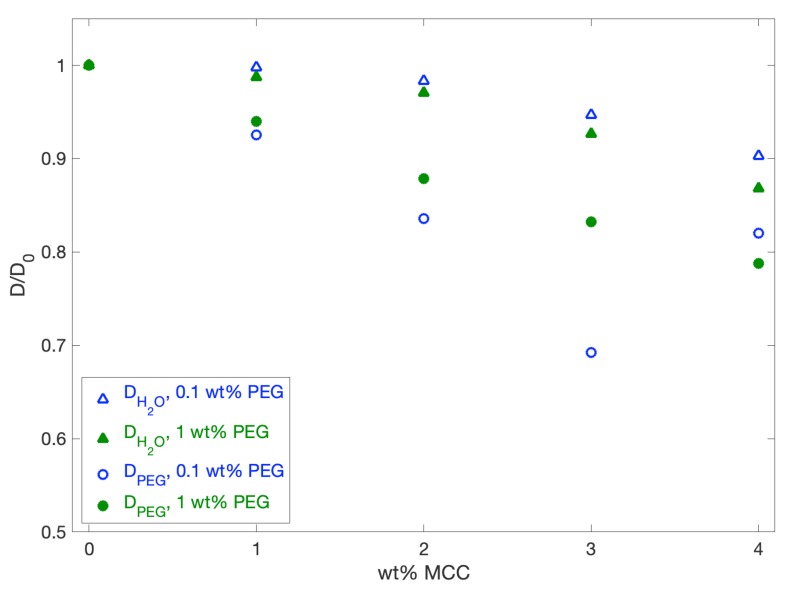
Diffusion coefficients $D_{H_{2}O}$ and $D_{\mathrm{PEG}}$ for H_2_O and PEG, respectively. The diffusion coefficients were measured at different MCC concentrations and divided by the diffusion in the neat solvent, $D_{0}$. Measurements were performed for 0.1 and 1 wt % PEG-200 (blue versus green data points).

To investigate possible interactions between PEG and cellulose, a NMR diffusion study was performed on PEG in neat NaOH versus in NaOH containing dissolved MCC. If PEG binds to MCC, the diffusion coefficient of PEG is expected to decrease, as MCC diffuses much slower than the small PEG molecules. The diffusion coefficients $D_{H_{2}O}$ and $D_{\mathrm{PEG}}$ of H_2_O and PEG, respectively, were measured at different concentrations of MCC and divided by the diffusion in the neat solvent, $D_{0}$ (Figure 8). The obtained data suggest a weak attraction, since the diffusion of PEG in the presence of MCC decreased more than the diffusion of water in the samples (open circles versus crosses, Figure 8). The diffusion of water did decrease slightly for increasing concentrations of MCC, since water has a fast exchange with the –OH groups of cellulose. If the determined diffusion coefficient for PEG in 0.1 wt % PEG and 3 wt % MCC is considered as an experimental outlier, there is no significant difference between the decrease in diffusion for the two investigated samples of 0.1 wt % and 1 wt % PEG (blue versus green curves, Figure 8). The fraction of PEG bound to MCC, $P_{b}$, can be expressed as

(11) $$P_{b}=1-\frac{D}{D_{0}}.$$

According to the obtained data, this implies that approximately 20% of PEG was bound to MCC at a concentration of 4 wt %. The fact that the $P_{b}$ values were similar for 0.1 and 1 wt % PEG is expected for weak adsorption, far from saturation. In a simple picture of Langmuir’s adsorption isotherm, this is consistent with the linear low concentration regime, where the adsorbed amount is proportional to the bulk concentration of the solute.

Previous studies reported in the literature seem to indicate that there is no interaction between PEG and cellulose in an aqueous state, although PEG has been found to associate with cellulose through hydrogen bonding if no water is present [30]. The determined interaction in the current study might thus indicate that an association between PEG and cellulose is also possible in an aqueous state, although the association is very weak, which suggests that considerably less hydrogen bonding occurs between PEG molecules and cellulose than in the dry state.

### 3.3. Exploring the Guiding Principles for Cellulose Solubility in Concentrated NaOH

Native cellulose is insoluble in water because of the very stable crystalline phase, often referred to as cellulose I. From the point of view of thermodynamics, this means that the transfer of cellulose molecules from the crystal state to water is associated with an increase in the Gibbs’ free energy, $\Delta G_{1}>0$. However, spontaneous dissolution can still occur if one combines the transfer with some second process that has a sufficiently negative $\Delta G_{2}$, so that the total free energy change of the two combined processes, $\Delta G=\Delta G_{1}+\Delta G_{2}<0$. One such second process, which has been utilized for over a century, is titration with a strong base. Sugars are weak acids, with p$k_{a}$ values around 12.5–13.5 [31]. When the pH > p$k_{a}$, the hydroxyl groups spontaneously deprotonate with the free energy change $\Delta G_{2}<0$. The higher the pH above p$k_{a}$, the more negative is $\Delta G_{2}$. This means that cellulose I should be soluble in an alkaline solution if the pH is sufficiently high, and the solubility of cellulose I should increase monotonically with increasing pH above p$k_{a}$. Experimentally, it has been found that 2 M NaOH is sufficient to dissolve some amounts of cellulose; 1 M NaOH is not sufficient.

However, increasing the NaOH concentration above 2 M does not lead to an increased solubility of cellulose. Instead, another instability sets in. The sodium salt of (deprotonated) cellulose also forms very stable crystalline phases with low solubility. Here, we need to consider the precipitation reaction cellulose${}^{n-}+n$ Na^+^ –> Na${}_{n}$-cellulose, with the solubility product K${}_{s}={\left[{\mathrm{Na}}^{+}\right]}^{n}\;\left[{\mathrm{cellulose}}^{n-}\right]$, and where the valency *n* can be a very large number. This results in a decreased cellulose solubility with increasing NaOH concentration. Thus, the solubility experiences two instabilities. At low pH it is unstable relative to native cellulose I, while at higher pH it is unstable with respect to the sodium salt, Na${}_{n}$-cellulose. Experimentally, it is found that cellulose can be somewhat dissolved in 2 M NaOH, but not in 1 M and not in 3 M. The small cation Na^+^ fits well into the dense crystalline lattice of cellulose, resulting in a stable crystalline phase. However, if we replace Na^+^ with a more bulky organic cation, such as tetrabutyl ammonium, TBA^+^, which does not fit into the crystalline lattice, then the salt crystal becomes unstable and large amounts of cellulose can be solubilized [16].

That organic bases are superior relative to NaOH in solubilizing cellulose has also been known for a long time. Still, however, they have not been shown to be commercially viable. Instead, a lot of research have been invested over the years into finding suitable additives to improve the solubility of cellulose in NaOH [5]. As discussed above, the effect of the bulky cation TBA^+^ is to destabilize the salt crystal state. Successful additives, instead, need to stabilize the solution phase. Many additives have been tested over the years, and in several cases improved solubility has been reported, although no dramatic improvement has been detected [5,10,27,28,29,32]. The experimental findings in the current study support this overall picture, although the addition of ZnO reduced the amount of aggregates present for samples containing 4 wt % MCC.

The question is then: What can be the action of an additive that leads to a decrease of cellulose’s chemical potential in solution and help “bind” it there? Additives that experience attractive interactions with the cellulose chains, alternatively with the Na^+^ counter-ions, have the potential to stabilize the solution phase. Binding the Na^+^-ions in solution can be practically difficult because of the high concentration (2 M), although one can of course imagine the action of complexing agents. Concerning the cellulose chain, Lindman and co-workers have in a number of recent papers pointed out its amphiphilic nature [6,7], which has been further debated in Glasser et al. [8]. In addition to its hydrogen bonding ability, it is suggested that there are also domains where the interaction with water (hydration) is less favorable. Additives or co-solvents that have an affinity for cellulose may improve solvation or form associations with cellulose that in both cases may increase the stability of the solution phase. The actions discussed above may work as guiding principles. In practice, the question is whether it is possible to generate sufficiently attractive interactions. Furthermore, strong attractive interactions may lead to associative phase separation [33].

## 4. Conclusions

In conclusion, the current study sheds further light on the dissolution properties of cellulose in aqueous NaOH. SAXS and SLS were employed in order to assess and compare different concentrations of MCC and the effects of additives. Scattering data revealed that it was possible to molecularly dissolve up to 2 wt % MCC in 8 wt % NaOH, which is contrary to previous findings. The obtained data suggest that the dissolved cellulose molecules can be characterized as semi-flexible cylinders with relatively long, stiff segments. This indicates that the chains are sterically hindered, most likely due to either solvent interactions or folded chain conformations.

SAXS data obtained in the current study furthermore revealed that the addition of ZnO reduced the aggregation level compared to cellulose in neat NaOH. Based on the observed levels of aggregation, the current study indicated that an optimal ZnO concentration for 4 wt % MCC in 8 wt % NaOH is approximately 0.7 wt % ZnO. The addition of PEG-200 did not improve the dissolution properties significantly, although the included diffusion NMR study did indicate a weak attraction between cellulose and PEG. The observed aggregation levels were slightly reduced for samples containing 0.1 wt % PEG, but the aggregation was increased for samples with a higher PEG content. Thus, the current study suggests that PEG most likely has no significant positive effect on the dissolution properties of cellulose in NaOH, at least not when following the sample preparation protocol employed in this study.

## Supplementary Materials

The following is available at https://www.mdpi.com/2073-4360/12/2/342/s1. Figure S1: Scattering Data for 2 wt % MCC in 8 wt % NaOH w/wo Additives.
