# Supplementary material for: Revisiting the Dissolution of Cellulose in NaOH as “Seen” by X-rays
Source: Polymers (Basel). 2020 Feb 5;12(2):342. doi: 10.3390/polym12020342 (PMC7077394; doi:10.3390/polym12020342)
Supplement: Supplementary file 1 [file polymers-12-00342-s001.pdf]

polymers

Supplementary Materials

Revisiting the Dissolution of Cellulose in NaOH as "Seen" by X-rays

Scattering Data for 2 wt% MCC in 8 wt% NaOH w/wo Additives

The scattering patterns for 2 wt% MCC in 8 wt% NaOH with ZnO or PEG is comparable to the scattering pattern of 2 wt% MCC in neat NaOH in the full *q*-range, which further supports the fact that MCC is completely dissolved at this concentration (Figure S1).

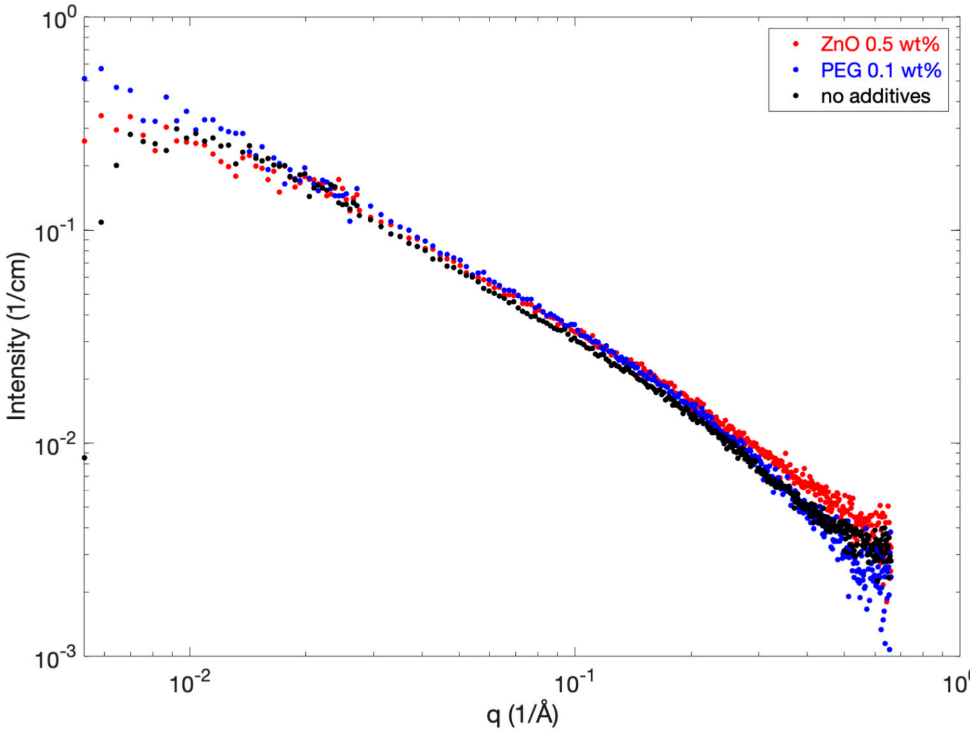

Figure S1. Comparison of SAXS patterns for 2 wt% MCC dissolved in 8 wt% NaOH, 8 wt% NaOH with 0.5 wt% ZnO and 8 wt% NaOH with 0.1 wt% PEG.

© 2020 by the authors. Licensee MDPI, Basel, Switzerland. This article is an open access article distributed under the terms and conditions of the Creative Commons Attribution (CC BY) license (http://creativecommons.org/licenses/by/4.0/).
